# Dynamics of *Tunga penetrans* infections and severity of associated morbidity among pigs during the dry season in rural Uganda

**DOI:** 10.1186/s13071-025-06716-z

**Published:** 2025-02-21

**Authors:** Francis Mutebi, Georg von Samson-Himmelstjerna, Hermann Feldmeier, Norbert Mencke, Charles Waiswa, Jürgen Krücken

**Affiliations:** 1 School of Veterinary Medicine and Animal Resources, College of Veterinary Medicine, Animal Resources and Biosecurity, Makerere University, Kampala, Uganda; 2 Institute for Parasitology and Tropical Veterinary Medicine, Freie Universität Berlin, Berlin, Germany; 3 Veterinary Centre for Resistance Research (TZR), Freie Universität Berlin, Berlin, Germany; 4 Institute of Microbiology, Infectious Diseases and Immunology, Charité University Medicine, Berlin Campus Benjamin Franklin, Hindenburgdamm 30, 12203 Berlin, Germany; 5 Bayer Animal Health GmbH, Leverkusen, Germany; 6 Present Address: Vetoquinol S.A, 37 Rue de La Victoire, 75009 Paris, France

**Keywords:** Pigs, *Tunga penetrans*, Dry season, Abundance, Morbidity, Disease dynamics

## Abstract

**Background:**

Tungiasis is a neglected tropical disease which is common in impoverished communities. In sub-Saharan Africa, it is caused by female sand fleas, *Tunga penetrans*, and pigs are amongst the major domestic animal reservoirs. Depending on the environment, tungiasis occurs throughout the year or preferentially in the dry seasons. This study investigated changes in sand flea abundance and associated morbidity in pigs during a dry season.

**Methods:**

*Tunga penetrans* lesions were counted and staged in 35 pigs amongst 22 households with at least one affected pig. Five weekly examinations were performed per animal during a dry season. Enrolment of pigs into the study lasted 17 days and examination was performed for 43 days. The severity score for acute pig tungiasis (SSAPT) was determined for each visit. Generalised linear mixed models (GLMM) were fitted on an absolute time scale to understand factors influencing the changes in abundance of sand flea lesions and SSAPT.

**Results:**

The prevalence/abundance of tungiasis-associated lesions increased from 57.1%/median 1 lesion at baseline to 71.4%/median 11 lesions after 4 weeks. In parallel, the median SSAPT increased from zero to six. The GLMM analyses fitting negative binomial models to the lesion numbers revealed that time had a linear and a quadratic effect for the viable stages 2 and 3a, and all viable sand flea stages in general with maximal abundance of sand fleas on days 17–18, 33 and 35 from the beginning of the study, respectively. The model for the total number of sand flea lesions, which included dead and excoriated sand fleas, showed the same trend but the peak was not reached within the study period. The number of stage 3b lesions was unexpectedly low. The SSAPT increased linearly over time and was highly dependent on the initial number of sand fleas at enrolment.

**Conclusions:**

There were increasing intensities of sand fleas and SSAPT in domestic pigs during the dry season. The ensuing environmental contamination by off-host stages of *T. penetrans* increases the risk of transmission to other susceptible hosts, including humans.

**Graphical Abstract:**

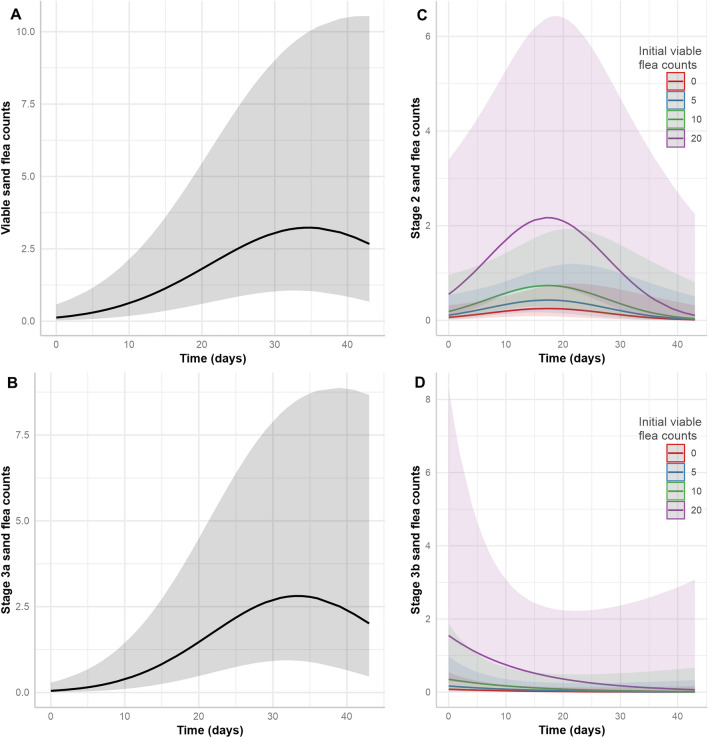

**Supplementary Information:**

The online version contains supplementary material available at 10.1186/s13071-025-06716-z.

## Background

*Tunga penetrans* infections are zoonotic and endemic amongst the poorest communities in Latin America and sub-Saharan Africa [1]. Tungiasis contributes to and worsens the poverty of affected communities [2] and was recently shown to affect the mental health of school children [3]. It has been mainly described in humans and 27 mammalian genera [4, 5] and was recently described amongst chicken in Brazil [6]. Animals act as reservoirs for *T. penetrans* and significantly contribute to the transmission and persistence of tungiasis in human populations [7, 8]. Available studies indicate that while dogs and cats are of major importance in tungiasis transmission cycle in Brazil [7, 9, 10], pigs are most significant as *T. penetrans* reservoirs in sub-Saharan African countries, including Uganda and Nigeria [8, 11]. Other important animal hosts include peri-domestic rodents and a wide range of wild mammals [7, 9, 10].

The life cycle of *T. penetrans* involves two phases; on-host tissue and off-host environmental development. Following penetration of the host skin, the 1 mm in length female sand flea rapidly increases to a size of a pea (by ~ 2000-fold in volume) in a period of about 2 weeks and remains embedded for another 2–3 weeks before it dies and is eliminated by the body [12]. The penetrated female sand fleas can be morphologically assigned to different developmental stages, that is, stage 2 lasting for 1–2 days after penetration and stage 3a present in the skin from approximately days 2–3 to days 5–6 after penetration. After days 6–7, stage 3b develops and lasts for approximately 2 weeks, during which a single flea lays up to 200 eggs [12]. After that, the fleas die in situ at stage 4. Although the infection is self-limiting, in endemic areas, reinfections are inevitable [13, 14] and all susceptible hosts can get infected independent of their age [8, 15, 16]. This implies that no protective immunity develops against reinfection with *T. penetrans* [2, 17]. Severe disease in people and animals results from a high infection intensity of *T. penetrans* [18–20], usually accompanied by secondary bacterial infections and excoriation of embedded fleas [21]. Severe infections lead to important sequelae, including septicaemia, gangrene, crippling body deformities and even death, which may be due to super infections including tetanus [21, 22].

Off host development of *T. penetrans* involves complete metamorphosis. Development from eggs to emergence of adult sand fleas takes between 14 and 31 days and requires dry, sandy and organic-matter-rich soils with no direct sun light [23]. Such conditions are usually most prevalent during the dry season in the tropical endemic areas. Indeed, in Brazil, the highest *T. penetrans* prevalence and intensities in human beings have been reported at the end of the dry season, while the lowest intensities were described during the rainy season [24]. However, the occurrence of tungiasis amongst Brazilian dogs appeared not to be significantly influenced by rainfall patterns [16]. Cross-sectional studies during the dry season in endemic villages of Uganda have reported high infection intensities and prevalence of *T. penetrans* amongst humans (up to 62.8%) [25] and domestic pigs (up to 64%) [8, 19]. However, infection dynamics of sand fleas during the dry season amongst pigs (the most important animal host for *T. penetrans* in Africa) in Uganda had never been established. Infection dynamics may influence the occurrence, severity and persistence of tungiasis, which in turn influence the planning and implementation of control measures. To obtain an insight into the pattern of *T. penetrans* transmission and development of severe tungiasis-associated morbidity amongst domestic pigs during a dry season when transmission rates are expected to be highest, infection intensities and tungiasis-morbidity were monitored over a period of 4 weeks in a highly endemic area. The study therefore demonstrated the increase in tungiasis-associated morbidity during the dry season and brought to light the animal welfare and potential economic implications of tungiasis.

## Methods

### Study area and study population

The study area was previously described in detail [8]. In brief, the study was conducted in ten neighbouring and socio-economically homogeneous villages drawn from three parishes in Bulidha sub-county of Bugiri district, Busoga sub region, south-eastern Uganda. The villages generally had a high prevalence of human and animal tungiasis, particularly amongst pigs. Pigs constituted a major livestock enterprise in the communities, though under poor animal husbandry practices, and their production was predominantly on subsistence scale. Pigs were reared on non-concrete (earthen) floors, were intermittently released to roam freely and control of ectoparasites was virtually non-existent. All pig-rearing households in the ten villages were targeted, but only those pigs from households with at least one infected pig were sampled.

### Study design

This was a longitudinal observational study undertaken from 16 January to 27 February 2015, a period which was characterised by dry weather conditions in Bugiri district and was considered to mark high transmission rates for *T. penetrans* [25]. All pig-owning households in the ten villages were identified and visited with the help of the respective Local Council One (LC 1) leaders. At household level, study objectives were explained to the pig owners and informed written consent was obtained.

### Diagnosis of tungiasis

For consenting households, a census of pigs was performed before pigs were examined systematically for tungiasis. Visibility of skin lesions caused by embedded female sand fleas on the digits was enhanced by thorough washing of the digits with a brush, soap and water. The entire body of the pig was clinically examined in a systematic order: head, trunk, tail, front and hind limbs by palpation and parting of hairs and observation to detect tungiasis lesions [26]. Lesions were staged according to the Fortaleza classification of the various stages of development of embedded sand fleas as described by Eisele et al. [12]. A dark spot of 1–3 mm often encircled by an erythematous zone (more readily detectable in pigs with a lightly pigmented skin) was characterised as stage 2, while a raised yellow to white or glassy nodule of 2–13 mm in diameter with a dark central spot characterised stage 3 lesions. Stage 3a lesions were firm and stretched the skin to form a convex-shaped white halo, while stage 3b lesions were shrunken and had a depression in the centre [12]. A brown-to-black circular patch often encircled by necrotic area is characteristic of stage 4 and an epidermal circular shallow crater characterises stage 5 lesions. Excoriated lesions caused by intense rubbing of affected sites against rough surfaces due to pruritus were also recorded.

### Inclusion and exclusion criteria

Only pigs from consenting households with at least one pig having at least one viable sand flea (Fortaleza stages 2 to 3b) were enlisted in the study. In addition to pigs being from a household with at least one pig with a viable lesion, pigs had to be apparently healthy (normal body temperature and no anaemia). Furthermore, only pigs which were not expected to leave the study area for a period of at least 3 months from the date of inclusion in the study were enrolled in the study.

### Recording of information for individual animals

For the enrolled pigs in each household (herd), data for all pigs were recorded on individual information forms and individual pigs were identified using ear tags. Individual pig data collected included: age, sex, breed, tungiasis-infection status, management system, herd size (number of pigs per household), household and village of enrolment. For infected pigs, sand fleas were counted, staged and associated morbidity described for each sand flea affected area. Sand flea localisation areas were photographed and the photograph serial numbers were recorded on the individual animal forms for review. Presence of any other ectoparasites was also recorded (positive/negative).

### Enrolment into the study and follow-up

At household level, pigs that met the inclusion criteria were allocated to two groups by simple random sampling using the lottery (fish bowl) method. One group was followed up for this study while the other group was allocated to another study arm (not described here). All pigs which were enrolled into the study were recruited, examined, cared for and followed up from their respective households. The pigs were being reared under a free-range management system. However, during the crop growing season, their movement would be restricted by tying them to trees or tree stumps near the human houses. Pig owners continued to look after the pigs in the same way they were before enrolment in the study. In case a pig was not examined at the time of the first visit, the household was revisited again on the same day or the following day or else it was excluded from the study. Owners were requested not to administer any form of treatment to the study pigs without consulting the investigator (F.M.) during the study period. Pigs within a household were all enrolled into the study on the same day. Overall, all pigs were enrolled into the study for a period of 17 days (from 15 January to 30 January 2015). A total of 28 pigs were enrolled in the first 7 days and the remaining seven pigs were enrolled in the next 10 days (day 8 to day 17 of the study). The day a pig was recruited into the study was considered as day 0 and thereafter, systematic examination of the pigs for tungiasis was done on a weekly basis (7 days’ interval) following recruitment for a duration of four weeks, that is, 0, 1, 2, 3 and 4 weeks. Although the study was envisaged to last for 8 weeks, it was terminated after 4 weeks on the basis of animal welfare considerations due to the rising infection intensities amongst the pigs. All infected animals were treated with Supona^®^ aerosol containing chlorfenvinphos 4.8%, dichlorphos 0.75% and gentian violet 0.145% (Pfizer, South Africa) at the end of the study [27].

### Assessment of the pigs during the study period

During the study period, the following parameters were assessed at every visit: the total number of embedded sand fleas, number of viable sand fleas (classified into the stages 2, 3a and 3b), number of stage 4 (dead) fleas, number of mutilated/excoriated flea lesions, number of body sites with at least one sand flea (described below) and the proportion of infected animals (%). In very severe cases, it was very difficult to delineate some lesions, and in such cases only the discernible lesions were included in the count for each body topographic site. Since the severity of tungiasis (SSAPT) [27] is directly proportional to the total number of sand flea lesions [13, 28], the SSAPT, modified from the severity score for acute tungiasis (SSAT) for humans [18], was used to assess the progression of the disease severity of the acute clinical manifestations of tungiasis. This was used as a complementary indirect assessment for increasing *T. penetrans* infection intensities amongst pigs. The modified morbidity severity score for acute tungiasis in pigs has been described previously [27].

Briefly, the SSAPT is a semi-quantitative assessment of the extent of the major acute clinical features of tungiasis on the predilection sites and their effect on the wellbeing of the pig. The clinical features considered are tungiasis-associated oedema, hyperaemia, ulcers, fissures, clusters of lesions, mutilation of lesions (as an indicator of itching), pain on digital pressure, ectopy of lesions, suppuration and/or abscessation and alteration of pig gait [27]. The SSAPT is a total sum of allocated scores to tungiasis lesion distribution on the body, and their effect on the animals’ wellbeing hence provides a good measure of morbidity accruing from the penetrated flea, damage due to lesion excoriations and secondary bacterial infections.

As the digits are the major penetration sites of sand fleas in pigs, they were used as the primary units for the scoring of the morbidity. The pig’s distal limb was divided into four topographic sites which included each of the principal and accessory digits up to the distal metacarpal or metatarsal joints. This makes a total of 16 digits (sites) for the four limbs of a pig. All other areas were considered ectopic infection sites for *T. penetrans*. Clinical signs were localised to each of the digits irrespective of extent and severity. Then a score of one to three was assigned depending on the number of body sites involved (Table 1). For other clinical manifestations which could not be attributed to individual sites, scores ranging from one to three were allocated depending on the relative significance of the sign on the wellbeing of the pig as proposed by Kehr et al. 2007 [18] for humans. For ectopic localisations, a score of 0.5 was allocated for every ectopic site for up to a maximum of eight ectopic sites. Therefore, the maximum SSAPT for an individual pig would be 27 (23 + 4).

**Table 1:** Summary of the computation of SSAPT (adapted from Mutebi et al.) [26]

Sign and number of sites affected	Score assigned
Hyperaemia and/or oedema^a^	
1–5	1
6–10	2
11–16	3
Pain on digital pressure	
1–5	1
6–10	2
11–16	3
Suppuration and/or abscess formation^a^	
1–5	1
6–10	2
11–16	3
Clustering of lesions^a,b^	
1–5	1
6–10	2
11–16	3
Fissure (s)^a^	
1–5	1
6–10	2
11–16	3
Skin ulcerations^a^	
1–5	1
6–10	2
11–16	3
Mutilation of lesions irrespective of sites involved (evidence of itching)	2
Altered gait/lameness	3
Ectopy of lesions (for each ectopic discrete body part involved up to a maximum of eight ectopic sites)	0.5

^a^Irrespective of number of foci and size of the area involved on a designated topographical site

^b^Three or more lesions in close proximity (1–2 mm apart)

### Data analysis

**Fig. 1:**
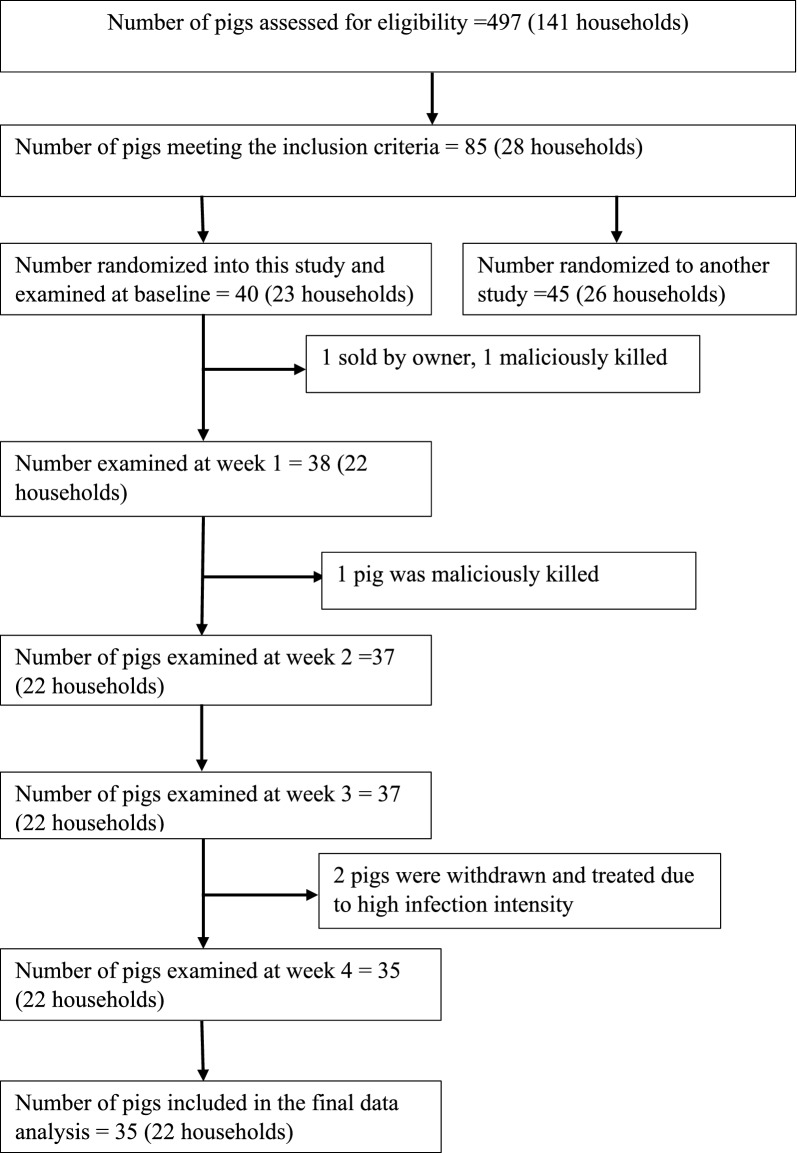
Flow diagram summarising the number of pigs followed up in each week following their enrolment in the study. Note that the pigs were enrolled over a period of 17 days (28 pigs within the first 7 days and seven in the following 10 days). The day of enrolment of a pig into the study was considered as day 0 and each pig was followed up weekly (7 days’ interval) for 4 weeks (28 days) from enrolment day

Only pigs which were examined on all occasions during the follow-ups were included in the final data analysis (Fig. 1). Data were analysed using R 4.3.3 [29] and GraphPad Prism, Version 5.03 for Windows (GraphPad Software, Boston, Massachusetts, USA). Initially data were entered into Microsoft Excel (2016) and verified for accuracy before exportation for analysis. The binomial test was conducted using the binom.test() function of R to determine whether male and female pigs were equally represented in the study population. Medians and interquartile ranges (IQR) were used to report centrality and dispersion of data, respectively. The 95% confidence intervals (CIs) for proportions (prevalence of tungiasis in pigs) were calculated using Wilson score intervals applying the binom.wilson() function from the epitools 0.5–10.1 package [30]. To identify significant differences between prevalences at different timepoints, the mid-*P* exact test was used as implemented in the tab2by2() function from epitools. A two-tailed Wilcoxon signed rank test comparing the median Spearman *ρ* coefficients for sand flea counts for the individual viable stages (Stages 2, 3a, 3b and 4) and the SSAPT calculated from individual pigs to a theoretical value of zero (no change over time) were conducted in GraphPad.

**Table 2:** Demographic information and management characteristics of the pigs in the study (*n* = 35)

Variable	Observation
Breed	All mixed breed
Village (number of pigs per village; %)	
Busindha	2 (5.7)
Masolya	9 (25.7)
Kibuye	15 (42.9)
Busakira	6 (17.1)
Namungodi	1 (2.9)
Busano	2 (5.7)
Sex (number; %)	
Male	14 (40)
Female	21 (60)
Age in months: median (IQR)	6 (4–12)
Herd size (number, %)	
1–4 pigs	20 (57.1)
5–7 pigs	15 (42.9)
Number of households/herds	22
Endo and ectoparasite control history	None

Data were analysed using generalised linear mixed models and the glmmTMB() function from the glmmTMB package 1.1.9 [31]. As dependent variables, the counts of different flea stages (2, 3a, 3b, 4) and the SSAPT were used in different regression models. As fixed factors in all regression models, the time (days after start of the study) as linear and quadratic effect, the sex, age and herd size were considered and three random affects were included in the initial models, that is, clustering on household levels, clustering on village level and random slopes over time. Optimisation of models was based on minimising the Akaike information criterion (AIC). The AIC estimates the error of prediction and the validity of the model of the parameter under investigation preferring models with high fit but penalising additional parameters in the model. Preferred models were those with a low AIC and for which scaled residuals from the data behaved similarly to simulated residues from the model as compared using the DHARMa package 0.4.6 [32]. Separate models for flea numbers were calculated for stage 2, stage 3a and stage 3b as well as for all viable flea stages (2 + 3a + 3b) and all flea lesions including dead stage 4 and excoriated lesions assuming a negative binomial distribution of the flea count data. For the SSAPT, a Gaussian distribution of the data was used as an approximation for the score data. The 95% confidence intervals (CIs) were calculated using the Wald method. The predictions of the models with 95% confidence bands were plotted using the ggpredict() function from the ggeffects 1.6.0 package. The SSAPT and the numbers of penetrated sand flea (stage 2, 3a, 3b, all viable and all flea lesions) per individual pigs were correlated to the study days to establish a Spearman’s rank correlation coefficients for each parameter and pig.

## Results

### Enrolment of pigs into the study

A total of 497 pigs from 141 households located in 10 villages and 3 parishes were evaluated for eligibility. Of these, 40 pigs from 6 villages and 2 parishes (Makoma and Wakawaka) were included in the study (Fig. 1). The pigs were recruited over a period of 17 days (Additional file 1: Table S1 containing raw data for the study). Overall, a total of 35 pigs from 22 households were examined in all the 4 follow-ups, and 5 pigs dropped out of the study due to various reasons (Fig. 1).

### Baseline parameters

The number of pigs enrolled from each household was variable (median = 4, IQR 3–6). The baseline demographic and management information of the pigs is summarised in Table 2. Overall, the majority of the pigs recruited in the study were female (60%) but the difference between the number of male and female pigs did not differ significantly (*P* = 0.31, exact binomial test).

The clinical characteristics of the pigs at the beginning of the study and the subsequent weeks are summarised in Table 3 (and more details are provided in Additional file 1: Table S1). While analysing these data, it was considered that the enrolment of pigs and their baseline examination was performed over a period of 17 days. For a study investigating increasing infection intensities of sand fleas in a specified period, this time lag is considerable and comparison of data based on specific study periods is not very meaningful. This is mainly because sand flea infection intensities start to increase with the commencement of the dry period and rapidly drop at the onset of the rainy seasons [24]. Accordingly, pigs which are enrolled earlier in the study may have lower numbers of sand fleas at baseline than those enrolled later, which may be attributable to this time lag.

**Table 3:** Summary of the parasitological and clinical characteristics of pigs throughout the study (*n* = 35)

Parameter	Timepoint				
	Baseline	Week 1	Week 2	Week 3	Week 4
Prevalence of tungiasis (%, 95% CI)	20/35 (57.1, 40.8–72.0)	20/35 (57.1, 40.8–72.0)	25/35 (71.4, 54.9–83.7)	24/35 (68.6, 52.0–81.4)	25/35 (71.4, 54.9–83.7)
Total number of lesions: median (IQR)	1 (0–5)	1 (0–10)	3 (0–21)	10(0–36)	11 (0–59)
Stage 2 + 3a: median (IQR)	0 (0–3)	1 (0–5)	2 (0–14)	7 (0–23	6 (0–35)
All viable lesions: median (IQR)	0 (0–4)	1 (0–5)	2 (0–15)	7 (0–24)	6 (0–35)
Number of sites with lesions: median (IQR)	1 (0–3)	1 (0–5)	2 (0–5)	3 (0–7)	4 (0–8)
SSAPT: median (IQR)	0 (0–5)	1 (0–6)	3 (0–6)	5 (0–7)	6 (3–8)
Number infested by other parasites (%)	29/35 (82.9)	33/35 (94.3)	33/35 (94.3)	32/35 (91.4)	34/35 (97.1)

**Fig. 2:**
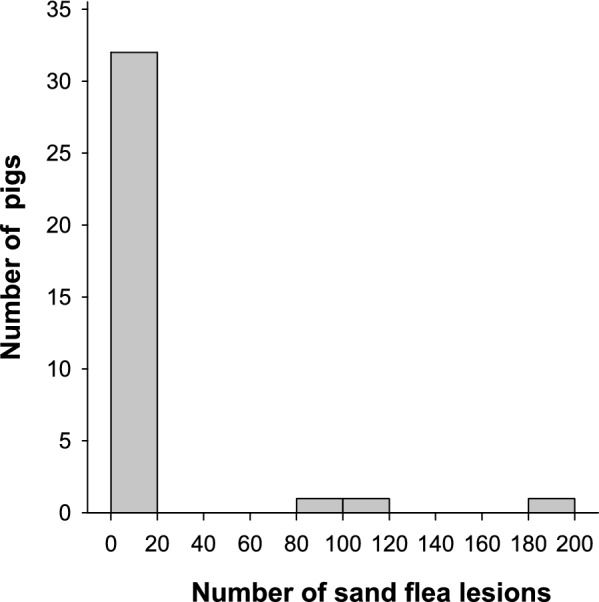
Histogram showing the distribution of sand flea lesions amongst the study pigs at baseline. Only three pigs carried 83% of all lesions

The prevalence of tungiasis amongst the pigs upon enrolment into the study was 57.1% (95% CI 40.9–72%; *n* = 20), which rose to a maximum of 71.4% (95% CI 54.9–83.7%; *n* = 25) in study week 4, but this increase was not significant (*P* = 0.226, mid-*P* exact test). On enrolment into the study, the majority of the pigs had less than 20 lesions (Fig. 2) and the median abundance was 1. Out of the total number of 474 viable and dead lesions amongst the 35 pigs, 394 (83.1%) were found on only 3 heavily infected pigs, all coming from the same household. A total of 10 of the 15 pigs which were not infected at baseline became infected in the course of the 4 weeks with a median sand flea intensity of 10 (IQR 3–11) amongst them by week 4. With the exception of one pig which also had lesions on the testes, all infected pigs had lesions only on the digits upon enrolment. By week 4, three pigs had lesions on other sites apart from the digits, that is, metacarpal and metatarsal (three pigs), testes (one pig) and snout (two pigs). Sand flea lesions were eliminated amongst 4 out of the 20 (20%) pigs which were infected at baseline in the 4 weeks of the study. These pigs had low sand flea intensities (1–5 sand flea lesions) at the beginning of the study.

Although sand fleas are hematophagous and their penetration is associated with secondary bacterial skin infections, neither gross anaemia (pale mucous membranes) nor fever (measured body temperature range = 38.4–39.2 °C which was within the normal range for pigs of 38.7–40.0 °C) were detected during the follow-up period in any of the pigs despite high sand flea intensities and suppuration of lesions in some infected pigs. As the study progressed, the abundance of the sand fleas increased and many lesions were excoriated by the pigs, making it very difficult to differentiate excoriated/mutilated lesions from those that had shrivelled at the end of their on-host life cycle. In addition, many newly penetrated sand fleas were localised at already infected sites, making it difficult to count all the lesions or detect new lesions in heavily infected pigs.

### Sand flea abundance and morbidity over time

**Fig. 3:**
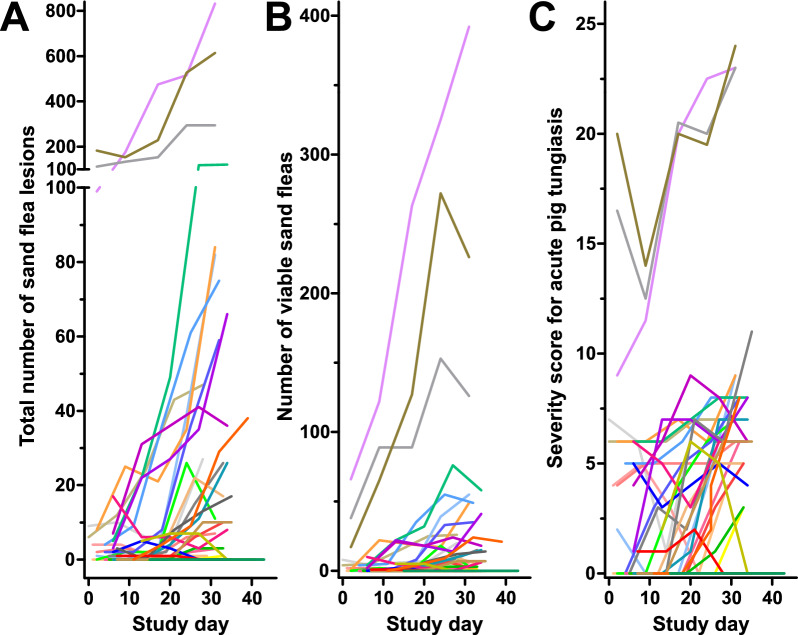
Time course of total number of sand flea lesions including excoriated lesions and stage 4 sand fleas (**A**), viable sand flea counts (stage 2 + 3a + 3b) (**B**) and severity score for acute pig tungiasis (SSAPT) (**C**). Each animal is shown in a different colour

**Fig. 4:**
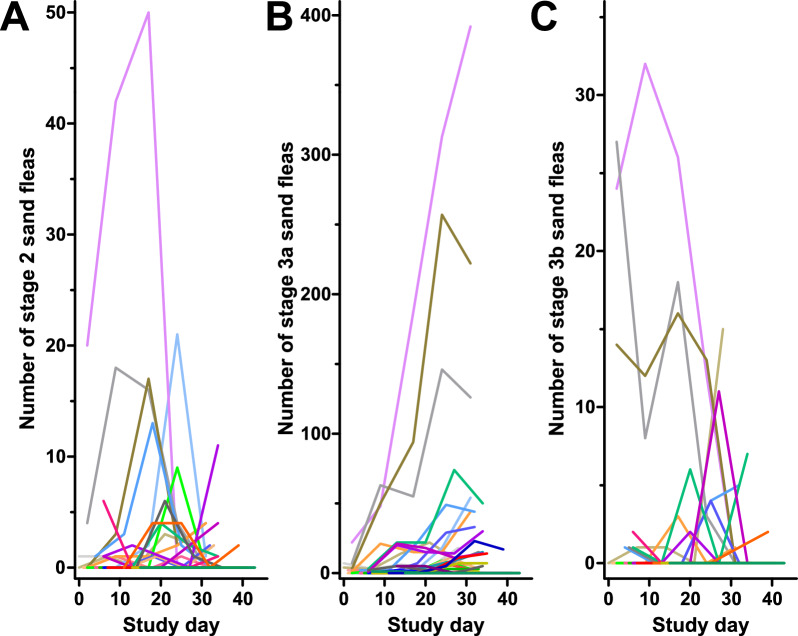
Number of the different stages of sand fleas during the study period in pigs. Time course of stage 2 (**A**), stage 3a (**B**) and stage 3b (**C**). Each animal is shown in a different colour

The time course of infection in the 35 individual pigs for which data were available for all timepoints is shown in Fig. 3 and Fig. 4. Although the large number of individual plots in the graphs makes it difficult to directly follow the data for individual pigs, the figures clearly show that there was a tendency for increased values over time. However, data for some individual animals also followed different trends against the overall trend, and in general the variability between animals was very high. Photos of the claws of two heavily infected pigs are shown in Additional file 2: Fig. S1. Due to the fact that pigs were enrolled into the study over a time period of 17 days and it was aimed at establishing the pattern of sand flea intensities in pigs during the dry season, data were analysed on an absolute time scale with day 0 fixed as the day when the first pig was included in the study. Data regarding total numbers of viable embedded female fleas (sum of the viable stages 2, 3a and 3b), total number of sand flea lesions and SSAPT are shown in Fig. 3. In addition, Fig. 4 shows the time course for the viable stages 2, 3a and 3b separately. Stage 3a was by far the most frequently found viable sand flea stage on the pigs followed by stage 2 and then stage 3b (compare scale of *y*-axes between Fig. 4A, B and C). There was an apparent increase of flea counts over time, but several individual pigs also showed a drop in flea counts at the end of the observation period. This was particularly the case for the total number of viable fleas (Fig. 3A) and the number of stage 3a fleas (Fig. 4B). For stage 3b, flea counts were very low at the end of the study (Fig. 4C). Data for the SSAPT showed more up and downs over time, but at the end of the study the majority of the pigs showed increased SSAPT values (Fig. 3B).

**Fig. 5:**
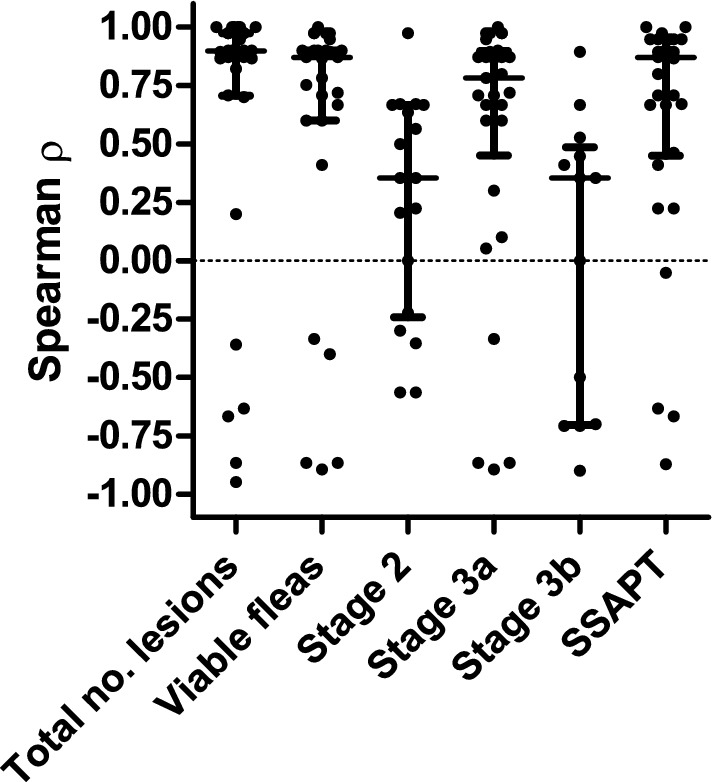
Spearman *ρ* correlation coefficients for total viable sand flea counts in pigs, sand flea counts for the individual viable stages and the severity score for acute pig tungiasis (SSAPT). Data show Spearman *ρ*-values for individual pigs, medians and interquartile ranges. Data for individual pigs are missing if all values for a pig were identical (e.g. if sand flea counts were zero). Data were compared with a hypothetical median value of zero using a two-tailed Wilcoxon signed rank test. ***P < 0.001; *P < 0.05; *ns* not significant

Since there were also pigs that showed strong decreases of total counts of sand flea lesions, viable sand flea counts including all stages individually or SSAPT over time and Spearman correlations for each parameter against study day were calculated for each pig individually (Fig. 5). Due to the small number of data points per animal (only five), many of these correlation analyses were not statistically significant. Moreover, no Spearman correlation *ρ*-values were obtained for data for which all values were identical (for example, total number of sand fleas or number of sand fleas of a particular stage over the entire study period). The analysis indicates that sand flea numbers and SSAPT increased over time. Although for all six parameters there was a wide range of correlation coefficients (range from −0.90 to 1), the majority of Spearman *ρ*-values were clearly positive (Fig. 5). The median Spearman *ρ* were 0.90, 0.87 and 0.78 for total sand flea lesions, viable sand flea counts and stage 3a sand flea counts, respectively. For the SSAPT, a very high median *ρ* of 0.86 was also observed. For stage 2 and stage 3b lesions, a considerably lower but still clearly positive median Spearman *ρ*-value of 0.35 was found. For all variables except of stage 3b, the median Spearman *ρ*-values were significantly higher than zero in a two-tailed Wilcoxon signed rank test (Fig. 5).

### Regression analyses for the sand flea count data

Separate regression analyses were conducted for total number of sand flea lesions, viable sand flea counts as well as counts for stages 2, 3a and 3b lesions. In all models, a linear and a quadratic time component were at least initially included. Since random effects on household level explained much more of the variability than random effects on village level, only the household level was included in the final models. The parameters sex, age and herd size were considered but did not improve any of the models and were therefore excluded.

**Fig. 6:**
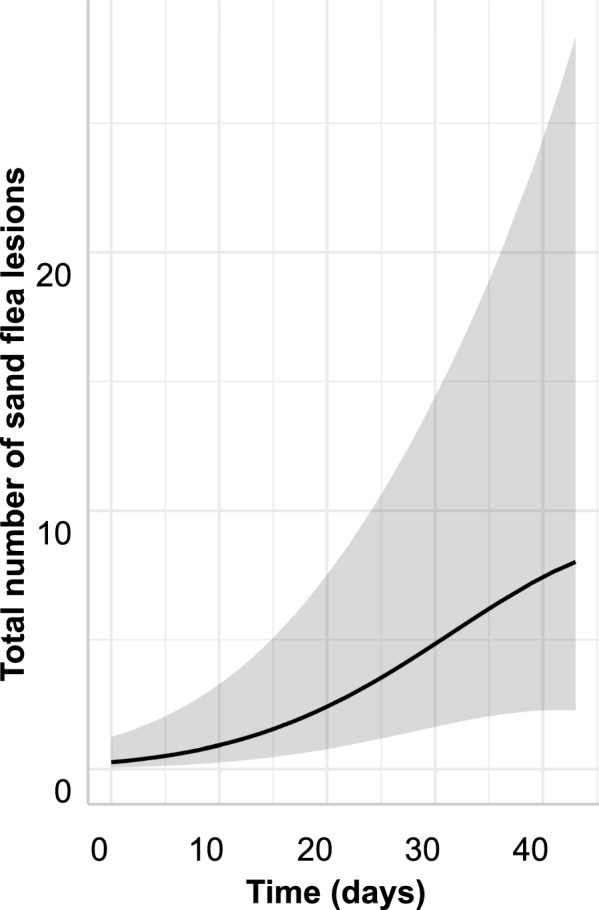
Graphical presentation of the effects of time on total number of sand flea lesions (including excoriated lesions and stage 4 sand flea lesions in pigs) predicted by the generalised linear mixed models specified in Tables 4. The shaded area shows the 95% confidence band

**Fig. 7:**
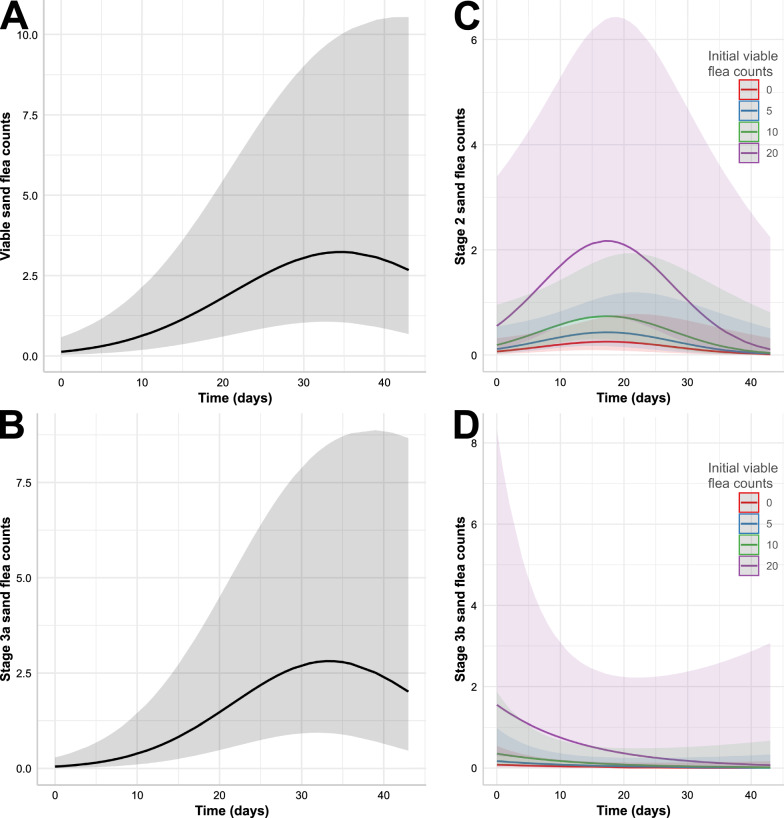
Graphical presentation of the effects of time and initial number of viable sand fleas on total viable sand flea counts (**A**) as well as counts of stage 3a (**B**), stage 2 (**C**) and stage 3b (**D**) lesions in pigs predicted by the generalised linear mixed models specified in Tables 4–7. Shaded areas show 95% confidence bands. The models presented in (**A**), (**B**) and (**C**) contain the time as linear and quadratic factor, while the model in (**D**) contains only the linear term. The models in (**B**) and (**D**) also show dependence of the initial number of viable sand fleas on the day a pig was enrolled in the study

The models for total number of sand flea lesions, total viable sand flea counts and stage 3a counts are detailed in Tables 4, 5 and 6, respectively. The final models only contained the time with a linear and a quadratic term as fixed effects. For the total number of sand flea lesions, the conditional coefficient of determination (*R*^2^) value was very high with 0.948 but the fixed effects explained only 9.3% of the variation (marginal R^2^ = 0.093) and the majority was explained by animal and household IDs (Table 4). There was a strong linear effect of time with an adjusted RR (aRR) of 1.15. However, there was also a highly significant negative quadratic effect of time, which leads to a model with decreasing slope towards the end of the observation period (Fig. 6). For the total viable sand flea counts and the stage 3a lesion counts, the positive linear effects were even slightly higher but the negative quadratic effects were also more pronounced (Tables 5 and 6). This led to optima of the sand flea count curves around approximately day 35 for total viable sand flea counts and day 33 for stage 3a lesion counts. For these models, the conditional R^2^ were again both > 0.9 while the effects of the fixed effects explained only 10–14% of the variation according to the low marginal *R*^2^ values. Figure 7A,B shows the prediction of the models for total viable and stage 3a sand flea counts graphically.

**Table 4:** Negative binomial generalised linear mixed model for count data on total sand flea lesions including excoriated lesions and stage 4 sand flea lesions in pigs

Variable	Effect type	aRR	95% CI	*P*-value^a^	*R*^2^
Intercept	Fixed	0.209	0.058–1.235	0.091	
Time	Fixed	1.147	1.083–1.214	< 0.001	
Time^2^	Fixed	0.999	0.998–0.9999	0.016	
Intercept animal ID	Random	2.444	n.a.	n.a.	
Time	Random	0.078	n.a.	n.a.	
Correlation intercept/time	Random	−0.961	n.a.	n.a.	
Intercept household ID	Random	2.420	n.a.	n.a.	
Conditional (all effects)					0.948
Marginal (fixed effects only)					0.093

^a^Determined using a *Z*-test

*aRR* adjusted rate ratio, *CI* confidence interval, *R*^2^, coefficient of determination, *n.a.* not available

**Table 5:** Negative binomial generalised linear mixed model for sand flea count data on total viable sand flea counts in pigs

Variable	Effect type	aRR	95% CI	*P*-value^a^	*R*^2^
Intercept	Fixed	0.124	0.0266–0.581	0.008	
Time	Fixed	1.207	1.124–1.296	< 0.001	
Time^2^	Fixed	0.997	0.996–0.999	< 0.001	
Intercept animal ID	Random	2.157	n.a.	n.a.	
Time	Random	0.077	n.a.	n.a.	
Correlation intercept/time	Random	−0.955	n.a.	n.a.	
Intercept household ID	Random	2.352	n.a.	n.a.	
Conditional (all effects)					0.915
Marginal (fixed effects only)					0.102

^a^Determined using a *Z*-test

*aRR* adjusted rate ratio, *CI* confidence interval, *R*^2^ coefficient of determination, *n.a.* not available

**Table 6:** Negative binomial generalised linear mixed model for count data on stage 3a sand flea lesion counts in pigs

Variable	Effect type	aRR	95% CI	*P*-value^a^	*R*^2^
Intercept	Fixed	0.050	0.009–0.290	< 0.001	
Time	Fixed	1.274	1.665–1.393	< 0.001	
Time^2^	Fixed	0.996	0.995–0.998	< 0.001	
Intercept animal ID	Random	2.666	n.a.	n.a.	
Time	Random		n.a.	n.a.	
Correlation intercept/time	Random		n.a.	n.a.	
Intercept household ID	Random		n.a.	n.a.	
Conditional (all effects)					0.911
Marginal (fixed effects only)					0.140

^a^Determined using a *Z*-test

*aRR* adjusted rate ratio, *CI* confidence interval, *R*^2^ coefficient of determination, *n.a.* not available

The model for stage 2 sand flea counts presented in Table 7 and Fig. 7C also shows a time effect with a negative quadratic and a positive linear time term. In comparison with the total viable sand flea counts, the peak of stage 2 sand flea counts was already on approximately day 17–18 days after start of the study and this was followed by a rapid decline in stage 2 lesion counts. The stage 2 sand flea count model also contains an additional fixed effect variable, that is, the initial viable sand flea counts on the day a pig was enrolled in the study had a strong positive effect on stage 2 sand flea lesion counts in the following visits (Fig. 7C). Due to the much smaller number of stage 2 sand fleas, the model suffers from higher unreliability. The conditional *R*^2^ of 0.768 suggests that there was a considerable number of effects that were not included in the model that had an influence on the stage 2 sand flea lesion counts. Remarkably, the marginal *R*^2^ describing the part of the variation explained by the fixed effects was 0.262 and thus considerably higher than for the model for total number of viable sand flea counts, which was only 0.102.

**Table 7:** Negative binomial generalised linear mixed model for stage 2 sand flea counts in pigs

Variable	Effect type	aRR	95% CI	*P*-value^a^	*R*^2^
Intercept	Fixed	0.063	0.013–0.318	< 0.001	
Time	Fixed	1.172	1.009–1.361	0.037	
Time^2^	Fixed	0.995	0.991	0.999	
Initial number viable fleas	Fixed	1.114	1.055–1.177	< 0.001	
Intercept animal ID	Random	0.184	n.a.	n.a.	
Time	Random	0.084	n.a.	n.a.	
Correlation intercept/time	Random	1.000	n.a.	n.a.	
Intercept household ID	Random	0.539	n.a.	n.a.	
Conditional (all effects)					0.768
Marginal (fixed effects only)					0.261

^a^Determined using a *Z*-test

*aRR* adjusted rate ratio, *CI* confidence interval, *R*^2^, coefficient of determination, *n.a.* not available

The model for stage 3a sand flea lesions (Table 6) is very similar to the model for total viable sand flea counts (Table 4), again containing only time as linear and quadratic terms. With an aRR of 1.207 for the linear effect, the effect of time is even slightly higher in this model compared with the model for all viable sand fleas. The model again shows an increase until approximately day 33 followed by a decrease (Fig. 6C). The conditional *R*^2^ was similar to that for the model for total viable sand flea counts, while the marginal *R*^2^ was slightly higher (0.140).

The model for stage 3b sand flea lesions was completely different from the model for earlier stages (Table 8, Fig. 7D). As already stated above, the number of stage 3b sand flea lesions was very low towards the end of the study. In the model, this leads to a negative but non-significant trend of stage 3b sand flea counts over time (aRR 0.930, *P* = 0.169). In contrast to time, the influence of the initial number of viable sand fleas on a pig was highly significant and had a positive impact of the counts for stage 3b lesions (Fig. 7D).

**Table 8:** Negative binomial generalised linear mixed model for the number of stage 3b sand flea lesions in pigs

Variable	Effect type	aRR	95% CI	*P*-value^a^	*R*^2^
Intercept	Fixed	0.080	0.012–0.544	0.010	
Time	Fixed	0.930	0.838–1.031	0.169	
Initial number viable fleas	Fixed	1.160	1.084–1.241	< 0.001	
Intercept animal ID	Random	0.905	n.a.	n.a.	
Time	Random	0.115	n.a.	n.a.	
Correlation intercept/time	Random	0.608	n.a.	n.a.	
Intercept household ID	Random	0.0001	n.a.	n.a.	
Conditional (all effects)					0.756
Marginal (fixed effects only)					0.233

^a^Determined using a *Z*-test

*aRR* adjusted rate ratio, *CI* confidence interval, *R*^2^, coefficient of determination, *n.a.* not available

### Regression analyses for severity score for acute pig tungiasis (SSAPT)

**Fig. 8:**
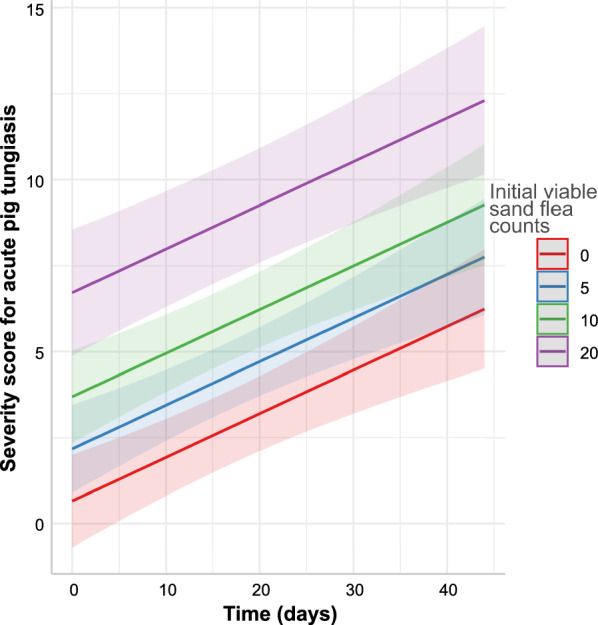
Effects of time and initial viable sand flea lesion count on the day a pig was enrolled on severity score for acute pig tungiasis

The SSAPT data were approximated using a gaussian generalised linear mixed model. The final model contained the time and the initial number of viable sand fleas at the time a pig was enrolled as highly significant linear terms (Table 9). The effects are graphically presented in Fig. 8.

**Table 9:** Gaussian generalised linear mixed model for severity score for acute pig tungiasis data

Variable	Effect type	*β* coefficient	95% CI	*P*-value^a^	*R*^2^
Intercept	Fixed	0.654	−0.693 to 2.001	0.342	
Time	Fixed	0.127	0.077–0.177	< 0.001	
Initial number viable fleas	Fixed	0.303	0.215–0.391	< 0.001	
Intercept animal ID	Random	3.542	n.a.	n.a.	
Time	Random	0.133	n.a.	n.a.	
Correlation intercept/time	Random	−0.582	n.a.	n.a.	
Intercept household ID	Random	0.0004	n.a.	n.a.	
Conditional (all effects)					0.916
Marginal (fixed effects only)					0.549

^a^Determined using a *Z*-test

*aRR* adjusted rate ratio, *CI* confidence interval, *R*^2^, coefficient of determination, *n.a.* not available

## Discussion

The occurrence and persistence of human tungiasis in endemic areas is influenced by a number of factors, such as available hosts, human behaviour and environmental conditions, which have been highlighted by a number of studies [15, 33–36]. Because many of these factors can be modified, they should be considered before designing strategies for tungiasis control. Pigs are considered to be one of the most important animal reservoirs of *T. penetrans,* at least in some sub-Saharan African countries [5, 8, 11, 25]. In many regions, human tungiasis is considered to be a disease showing strong seasonal variation with peaks of disease burden in the dry season [24]. However, data regarding the changes of abundance of sand fleas and disease burden over time in the dry season are largely missing. In the present study we used naturally infected domestic pigs in pig-keeping households to characterise the dynamics of infections as revealed by the abundance of early sand flea stages and the dynamics of disease burden as revealed by total sand flea counts and SSAPT as a measure of clinical impact.

Overall, data on the number of embedded sand fleas might be prone to several errors. The small numbers of stage 2 and stage 3b sand flea lesions can in particular be partially caused by confounders. Stage 2 lesions are smaller and are more readily detectable when associated with hyperaemia. On sites where the density of fleas is high and there are extensive abrasions or necrosis of the skin, stage 2 sand flea lesions may be obscured and not be readily detectable. Further, extensive mutilation of lesions with progression in segmental hypertrophy (neosomy) and therefore pruritus may contribute to excoriation of many sand fleas before they develop to stage 3b and therefore do not survive to complete their lifespan. These factors may have contributed to the low detection rate of stage 2 and 3b lesions, respectively, amongst the pigs. The excoriation/mutilation of lesions by the pigs before the completion of their lifecycle may also hinder the on-host monitoring of embedded sand flea development and viability amongst pigs, such as in studies evaluating the efficacy of medicines against sand fleas. In such cases evaluation of SSAPT would be an appropriate alternative or addition.

Infection rate would be the best outcome measure for new infections [13, 14] but it was not possible to count all embedded sand fleas at every examination. This was due to high intensities in some pigs and the high rate of excoriation of lesions amongst pigs with tungiasis-associated pruritus leading to rubbing of affected sites against hard objects or the ground [26]. In addition, sand fleas tend to penetrate already infected sites, making discernment of individual lesions in heavily infected animals difficult. Therefore, abundance of sand flea lesions, prevalence and morbidity scores were used in this study to evaluate the changes in sand flea transmission. Fortunately, these parameters are strongly correlated with infection rate [18].

Herein, sand flea infection intensities and morbidity amongst pigs strongly correlated with the time from the beginning of the study, which marked the beginning of the dry season, and as the dry season proceeded. However, this trend was highly heterogeneous and not observed for all individual pigs. Data were also highly clustered on the household level, which became immediately obvious since the three pigs which had the highest sand flea counts came from the same household. Therefore, linear mixed models with random effects variables were fitted using also random slopes for individual animals. The models for different sand flea stages and the SSAPT generally followed three different patterns. A linear and quadratic term was included in the models for stages 2, 3a, total number of viable sand fleas and total number of sand flea lesions. The quadratic term leads to curve shapes with a maximum; however, the kinetics for the different parameters were different for the different sand flea stages. For the earliest stage in the developmental cycle of the sand flea, that is, stage 2, the peak of the modelled curve was already reached between study days 17 and 18. For the next, stage 3a, the peak occurred considerably later at day 33. This was also the case for total viable sand flea counts, which were dominated by the high number of stage 3a, on day 35. For the total number of sand flea lesions including stage 4 and excoriated lesions the peak was not reached within the observation period. Considering the fact that the stages 2 and 3a lasted for 2 and 2–3 days, respectively, and are the earliest stages of the on-host part of the developmental cycle [12], their abundance most likely reflect the kinetics of transmission pressure during the dry season. The earlier and the more short-lived a stage is, the better it is suited to detect new infection events and estimate the incidence of infection. This is because they mark the recent establishment of a flea in the host. In contrast, later stages have a much longer life span and the time when an infection occurred can only be vaguely estimated. The early peak of stage 2 lesions suggests that already from study day 18, the infection pressure started to decrease. However, the number of stage 2 sand fleas that were found during the study was low in comparison with stage 3a and thus estimation of the model parameters is less precise. Further, the model for stage 3a showed a very similar pattern characterised by a decrease of abundance of penetrated sand fleas of this stage already before the end of the dry season and onset of rainfall. Such changes might be caused by some self-limiting effects. One such self-limiting effect is obviously a more severe itching with the increasing number of penetrated fleas leading to more intense rubbing of the pig digits and finally mutilation and excoriation of viable sand fleas. However, it cannot be entirely excluded that competition regarding resources occurs at high densities. These might include limited feed for larvae in the soil, cannibalism of larvae feeding on sand flea eggs, as is well known for cat fleas [37], or limited optimal penetration sites on highly infected pigs.

For the total number of lesions caused by sand fleas, the peak was not yet reached at the end of the study period. The stage 3b model did not contain a quadratic term and the linear effect of time on the number of stage 3b lesions was negative. Moreover, stage 3b was the stage that was the rarest of all viable stages. Both observations are highly unexpected since stage 3b lasts for about 2 weeks and thus roughly 5–7 times longer than the stages 2 and 3a. If the infection rate would be constant over time, the number of stage 3b lesions is expected to be 5–7 times higher than those of stages 2 and 3a. Further, if the number of penetrating sand fleas increases over time, as the data for stages 2 and 3a suggest, then stage 3b lesions might be lower than stage 3a lesions at the beginning of the season but stage 3b counts should increase over time. Since neither of these scenarios is close to the time course of observed and modelled stage 3b fleas, it must be assumed that stage 3b numbers are not driven by the infection rate and thus the environmental conditions. The obvious explanation is that development of stage 3a to stage 3b is limited by some mechanisms. Although the present study did not address the identification of mechanisms that prevent further development of early stages of sand fleas to the mature stage, the discrepancy between total number of sand flea lesions (including stage 4 and excoriated sand fleas) and number of viable sand flea lesions is obvious. Most likely, excoriation of the viable sand fleas by the pigs is quite efficient and a large proportion of living sand fleas does not progress to stage 3b. As suggested above, this might also be a density dependent mechanism since pain and itching are expected to increase with infection intensity, leading to increased rubbing of the affected parts against hard surfaces in the environment such as the ground or trees. Another density-dependent mechanism that cannot be excluded in pigs with very high sand flea burdens is that some sand fleas cannot complete development in very dense clusters or that the number of sand fleas in these clusters is simply underestimated by counting.

The initial sand flea counts at baseline were included in the final count models for stages 2 and 3b. This parameter can be interpreted as a proxy for previous sand flea exposure of the pig before the beginning of the study and that animals with initially high counts remained with higher counts throughout the study. Although this parameter might be at least partially covered in the random effects of the models (household and animal ID) for some of the models, this factor was kept in the models for stage 2 and 3b since it improved them in terms of AIC and distribution of residuals, suggesting that some of the animals from the same household behaved differently than others depending on the initial sand flea counts. One simple explanation can be that the infection pressure differed on the compound of households and when pigs are tethered to different trees on the compounds and stay in their places, they might be exposed to host-seeking *T. penetrans* to different degrees. The fact that the parameter was only included in the models for stages 2 and 3b, which both show an early peak, or continues to decrease and both rely on a small number of lesions, but was not included in the models showing a late peak and relying on a large number of sand flea lesions, is also remarkable. In the models for stage 3a, all viable sand fleas and all sand flea lesions, the early timepoints were generally less influential for the estimation of the model parameters than in the models for stages 2 and 3b, which can explain why initial sand flea counts were only included in the latter. It is well known that female sand fleas tend to form clusters [22], which is also observed for other ectoparasites such as *Amblyomma variegatum* ticks [38]. This might facilitate the finding of embedded females by males since mating occurs between free males and penetrated females on the host [39]. If the same effect is also relevant for host finding and the chance that a female sand flea penetrates the skin of a host, this might contribute to the fact that high initial sand flea numbers attract more sand fleas. Furthermore, further infections may be facilitated by the skin damage caused by already-penetrated sand fleas and skin mutilation due to tungiasis-associated pruritus.

In contrast to the sand flea counts, the model for the SSAPT was fitted using a linear (Gaussian) model. The final model contained time as a linear factor but not a quadratic factor and in a Gaussian model this resulted in a simple linear increase of the severity score over time. In addition, the initial number of sand fleas at enrolment had a strong linear effect on the severity score. Since it has previously been shown that severity of tungiasis is correlated with the sand flea number [13, 28], the latter observation is not unexpected. Moreover, the SSAPT is calculated from the number of affected sites and the clinical symptoms and signs. Dead and excoriated lesions also contribute to disease severity. The rubbing of affected sites against hard objects not only leads to excoriation of the sand fleas, but also contributes to the severity of clinical signs and symptoms. The total number of sand flea lesions, including the dead stage 4 sand fleas and the excoriated sites, increased until the end of the observation time, and the continued increase in the severity score is a direct consequence of this. With a longer observation period, when the total number of sand flea lesions would decrease again, the SSAPT is also expected to decrease again, probably towards the end of the dry season and thus later than any of the numbers for sand fleas and sand flea stages that were determined in the study. However, the 4-week observation period per pig that was possible in the present study was not suitable to obtain data from this period of decreasing severity and it was therefore not possible to model it. Although it cannot be directly concluded from the data, it can be assumed that a prolonged dry season is likely to influence the occurrence of severe tungiasis amongst humans and animals. However, the fact that at least the numbers of early stages of sand fleas peaked already before the end of the observation period suggests that some self-limiting effects also play a role. It would be interesting to establish how the changes in animal infections correlate with environmental contamination by off-host sand flea stages and the occurrence of the infection in humans.

The study demonstrated for the first time a pattern of increasing sand flea abundance and tungiasis severity in domestic pigs in a single dry season using a quantitative approach and multiple visits within the same dry season. Tungiasis-associated morbidity amongst affected animals has various economic implications including low productivity and merchantability [5]. The study has demonstrated that amongst pigs from households with tungiasis, the abundance of sand fleas and sand flea lesions increase rapidly during the dry season. The increasing abundance contributes to increasing disease severity amongst affected pigs. The resultant heavy environmental contamination by off-host stages of sand fleas poses a risk for humans sharing home ranges with affected pigs. Thus pigs may contribute to amplification of *T. penetrans* infections. Therefore, control measures should be put in place early in the dry season to guard against the upsurge of tungiasis prevalence and intensity at an early timepoint to prevent an occurrence of a high disease burden in a short time frame.

The dry seasons mark the crop harvest time, and after harvest, pigs are released to roam freely with minimum restrictions [8]. Extensive animal management systems as found in the study area lead to an overlap between human and animal home ranges. This may also lead to contamination of previously unaffected areas with sand flea eggs from pigs that roam around between neighbouring households in the villages. Unfortunately, in many endemic communities, the occurrence of tungiasis amongst animals is not well known to animal owners [40, 41] and effective prophylaxis or treatment for animals and humans are also not accessible to affected communities [5, 27].

Amongst human populations in Fortaleza (Ceará state in the region Northeast of Brazil, with a semi-arid climate), tungiasis has a seasonal pattern with the highest prevalence and intensity occurring at the end of the dry season [24]. Further, the occurrence of tungiasis amongst Brazilian dogs in Ilhéus (Bahia state, also in the region Northeast of Brazil but approximately 1200 km south of Fortaleza, but with a tropical climate) was not influenced by seasons and rainfall patterns [16]. This difference can be explained by the fact that Fortaleza has a substantial dry season from August to November while average precipitation is continuously high in Ilhéus throughout the year. This study has demonstrated that in Uganda the sand flea infection intensities amongst pigs increase during the dry season but it is yet to be demonstrated whether tungiasis or off-host stages of *T. penetrans* persist during the rainy seasons of the year in animals, humans or the environment in Uganda.

The increasing number of embedded sand fleas amongst the pigs suggests continuous infections during the dry season and a continuous increase in the number of infectious stages in the environment at least for the beginning of the dry season. In such a setting, repeated treatments are not practical and are expensive. The exception would be using systemic and long-acting prophylactic compound formulations such as the recently introduced isoxazolines or bispyrazoles [42–44] during the dry season. The fact that a long-acting product containing the isoxazoline fluralaner is now on the Brazilian market for cattle [45] suggests that it might be possible to also develop such a strategy for pigs. However, there remains obvious challenges for people practising subsistence farming, such as high product prices and correct application of withdrawal times for long-acting systemic compounds. In the absence of such formulations registered for pigs, environmental sanitation and pig confinement remain the most appropriate tungiasis control practices in such settings [13].

One of the limitations of the study was that it had a limited time scope. The entire study lasted for only 6 weeks but each pig was followed up for 4 weeks during the dry season. In addition, the study involved only a small number of pigs. A longitudinal study involving examination of a bigger number of humans and animals during the various seasons of the year would give a better understanding of the infection dynamics of *T. penetrans* in endemic villages. This can be complemented by analysis of soil samples in both human and animal dwellings for off-host stages of *T. penetrans* [46]. Future studies should also investigate the effect of environmental factors, especially temperature and relative humidity on the occurrence of off-host stages and therefore the kinetics of on-host infections.

## Conclusions

The study demonstrated that in pigs from rural tungiasis endemic villages of Uganda, the abundance of sand flea lesions increases rapidly during the dry season. Subsequently, the increasing abundance of sand fleas contributes to increasing disease severity amongst affected pigs. The resultant heavy environmental contamination by off-host stages of sand fleas presents a risk for people sharing home ranges with affected pigs. Thus pigs may contribute to amplification of *T. penetrans* infections in endemic communities.

## Supplementary Information

Additional file 1: Table S1: Raw data for all pigs included in the study.

Additional file 2: Fig. S1. Development of abundance of penetrated sand fleas in two selected severely infected pigs from week 0 to week 4.

## Data Availability

All relevant data have been included in the manuscript and its supplements.

## Abbreviations

AIC: Akaike information criterion
CIs: Confidence intervals
CoVAB: College of Veterinary Medicine, Animal Resources and Biosecurity
GLMM: Generalised linear mixed models
ID: Identity
SSAPT: Severity score for acute pig Tungiasis
