# Supplementary figures and images for: Dynamics of Tunga penetrans infections and severity of associated morbidity among pigs during the dry season in rural Uganda
Source: Parasit Vectors. 2025 Feb 21;18:64. doi: 10.1186/s13071-025-06716-z (PMC11843770; doi:10.1186/s13071-025-06716-z)

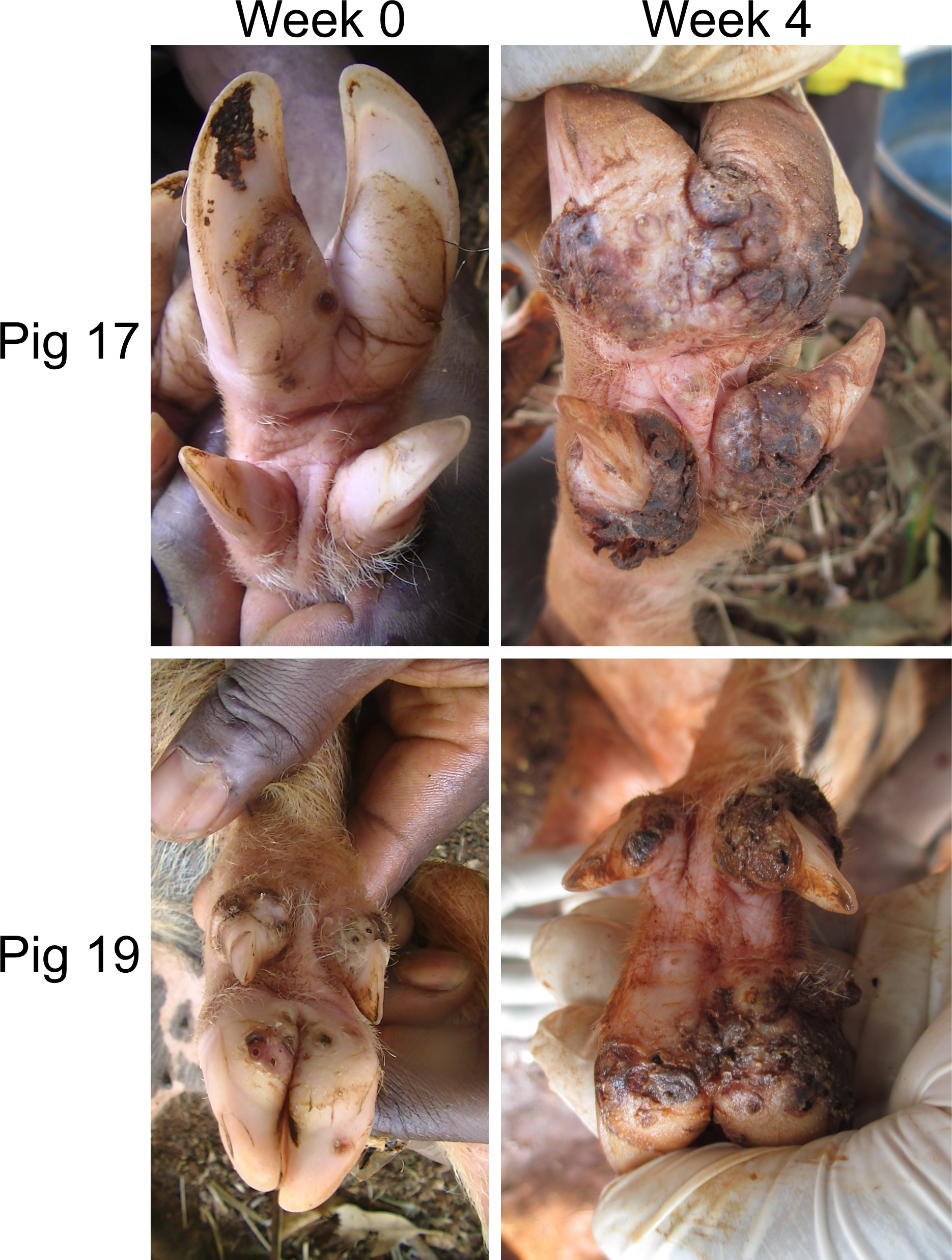

Supplement: Supplementary file 2 — Additional file 2: Fig. S1. Development of abundance of penetrated sand fleas in two selected severely infected pigs from week 0 to week 4. [file 13071_2025_6716_MOESM2_ESM.jpg]
